# Mechanism of Oxygen–Chlorine Potential Interaction During the Ca/Y-Mediated Solid-State Deoxidation of Zirconium

**DOI:** 10.3390/ma19122515

**Published:** 2026-06-11

**Authors:** Zhaohui Ma, Guoqing Yan, Xiao Yan, Haiyang Ding, Gang Wu, Shunli Zhang, Jiandong Zhang, Weidong Chen, Lijun Wang

**Affiliations:** 1 Zhongyuan Critical Metals Laboratory, Zhengzhou University, Zhengzhou 450001, China; mazhaohui@grinm.com (Z.M.); weidongch@163.com (W.C.); gold@grinm.com (L.W.); 2 National Engineering Research Center for Environment-Friendly Metallurgy in Producing Premium Non-Ferrous Metals, Beijing 101407, China; yanxiao@grinm.com (X.Y.); wugang@grinm.com (G.W.); zhangshunli@grinm.com (S.Z.); zhangjiandong@grinm.com (J.Z.); 3 GRINM Resources and Environment Tech. Co., Ltd., Beijing 101407, China; 4 College of Materials Science and Engineering, Inner Mongolia University of Technology, Hohhot 010051, China; dinghaiyang108@icloud.com

**Keywords:** external gettering, zirconium, oxygen–chlorine potential interaction, Ca/Y-mediated deoxidation

## Abstract

**Highlights:**

- Dynamic shift of oxygen mediator from Ca to metallic Y by increasing chlorine potential, explaining non-monotonic O variation in Zr.
- Establishment of three-phase equilibrium (Y–YOCl–YCl_3_) enabling in-depth deoxidation of Zr down to 20 ppm at 1173 K.
- Synergistic mechanism of O and Cl potentials clarified, providing a novel framework for designing efficient gettering systems.

**Abstract:**

Zirconium (Zr) is a strategic metal resource whose performance is significantly degraded by high oxygen content. The external gettering process is an effective approach for in-depth deoxidation of Zr. In this study, the deoxidation behavior of Zr in the Ca-Y-CaCl_2_ external gettering system was investigated by adjusting the chlorine potential through YCl_3_ addition. The change of oxygen potential and its synergistic control mechanism during the variation of chlorine potential were systematically examined. The results demonstrated that with increasing chlorine potential, the system undergoes a sequence of reactions: chlorination of Ca, formation of metallic Y, formation of YOCl, dissolution of Y_2_O_3_, and formation of YCl_3_, ultimately reaching a three-phase equilibrium of Y-YOCl-YCl_3_. During this process, the oxygen content of Zr fluctuates notably, which is primarily attributed to the shift in the oxygen-transfer medium from Ca to Y. This transition changes the oxygen potential control mechanism from indirect Y-Ca control to direct Y control. After reaching equilibrium at 1173 K for 72 h, the equilibrium oxygen content of Zr initially remains stable with increasing chlorine potential, then gradually decreases, eventually reaching 20 ppmw. This trend is consistent with the mutual interaction of oxygen potential and chlorine potential. The findings provide important theoretical insights into the interaction between oxygen and chlorine potentials in deoxidation systems, elucidate the multi-element synergistic mechanism for oxygen control, and contribute to the design of efficient deoxidation systems.

## 1. Introduction

Zirconium (Zr) and its alloys have garnered significant attention in the nuclear, optical, and electronic industries owing to their exceptional combination of physical and chemical properties [1,2,3,4,5]. Key attributes include a high melting point, outstanding corrosion resistance, superior glass-forming ability, favorable machinability, and a low thermal neutron absorption cross-section [6,7,8]. Furthermore, high-purity Zr serves as a vital precursor for fabricating ZrO_2_ films via vapor deposition, which exhibit excellent optical and electronic performance [9,10]. To meet the requirements of such demanding applications, the oxygen content of Zr materials must be maintained at a low level. High oxygen content can degrade material performance, leading to embrittlement and hardening of the matrix [11,12], reduced glass-forming ability [13], and splashing during evaporation coating processes, which may shorten service life and even pose safety risks [8,14,15]. Nevertheless, the strong affinity of Zr for oxygen, combined with its high oxygen solubility [16,17], limits the deoxidation efficacy of conventional high-purity refractory metal melting technologies—such as electron beam (EB) melting and vacuum arc (VA) melting—in achieving in-depth purification [13,14]. Iodide purification remains the predominant technique for deoxidizing Zr, capable of reducing oxygen content to below 100 ppmw. However, this process is constrained by several limitations, including fluctuations in oxygen content, high energy consumption, substantial metal loss, and inapplicability to fabricated materials. Consequently, effective control of oxygen contamination remains a formidable challenge in the production and processing of high-purity Zr materials.

**Figure 1:**
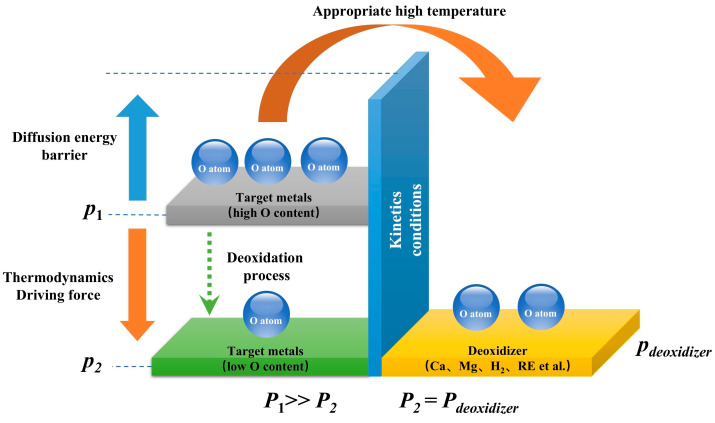
Schematic diagrams of the external gettering process.

External gettering is an effective deoxidation technique for Zr. Its fundamental principle involves two aspects, as depicted in Figure 1. (I) Thermodynamic driving force. A deoxidation system can be established by enclosing a target metal (e.g., Ti, Zr) together with a deoxidizer in a reaction vessel under a specific atmosphere. The deoxidizer absorbs oxygen within the system and reduces the oxygen potential (refers to the oxygen partial pressure (or its logarithm) corresponding to a given reaction or system). This creates an oxygen potential gradient between the system and the target metal, facilitating the diffusion and removal of oxygen. (II) Kinetic conditions. Appropriate kinetic conditions are also required. A suitably high temperature provides the necessary thermal energy for oxygen atoms to overcome the diffusion barrier, which is a prerequisite for diffusion to occur under the thermodynamic driving force [18].

In an external gettering deoxidation system, the deoxidizer is critically important, as it directly determines the deoxidation capacity. An effective deoxidizer must meet the following requirements: (I) possess a high oxygen affinity; (II) exhibit good chemical compatibility with the target metal and the crucible material; (III) remain physically and chemically stable under the deoxidation conditions. Currently, the most widely used deoxidizers are elements from the groups I A and II A, such as H_2_, Mg, and Ca [19,20,21,22,23]. Some elements from groups III A and IV, such as Al [24] and C [25], are frequently employed in metallothermic reduction processes. Some refractory metals from the transition groups, such as Ti, Zr and V can also serve as deoxidizers or getters [26,27,28,29].

Besides the above deoxidizers, rare earth metals (REs) have recently demonstrated promising potential for the deoxidation of refractory metals [30,31]. Compared to deoxidizers such as Ca and Mg, they exhibit significant advantages in enhancing the thermodynamic limit of deoxidation. REs generally have high oxygen solubility at elevated temperatures, readily forming RE-O solid solutions which possess lower oxygen potential than corresponding oxides according to Henry’s law. Even when oxygen is saturated, the RE-RE_2_O_3_ equilibrium can still maintain a relatively low oxygen potential. Further regulation of the chlorine potential (refers to the chlorine partial pressure (or its logarithm) corresponding to a given reaction or system) of the system can lead to the formation of oxyhalides (REOX, X: Cl or F) phases, which can create an ultra-low oxygen potential conducive to in-depth deoxidation.

Researchers have explored the efficacy of RE-based deoxidation. T.H. Okabe et al. [32,33] and J.D. Corbett et al. [34] systematically discussed the oxygen potentials corresponding to different phases of REs, such as Y, Ce, and La, in chloride and fluoride systems, from a thermodynamic perspective, providing a theoretical basis for constructing rare earth-based deoxidation systems. T.H. Okabe et al. [35] investigated the deoxidation effect of Y on Ti in a CaCl_2_ molten salt system. The oxygen content in Ti ranges from 10 to 80 ppmw when a Y-O solid solution is formed, while under Y-Y_2_O_3_ equilibrium conditions, the oxygen content increases to 90–720 ppmw. Iizuka et al. [36], L.N. Jiao et al. [37], Z.H. Ma et al. [38], C.L. Lu et al. [39], and Z.W. Huang et al. [40] investigated the deoxidation mechanism of Al-Ti alloys, Zr, La-Ce alloys, and Ti by Y or Y-Ca. These works collectively confirmed that Y can effectively reduce oxygen content in these metals to below 100 ppmw and highlighted the promising potential of combined Y–alkaline earth metal (e.g., Ca, Mg) systems for in-depth deoxidation of refractory metals.

Furthermore, significant advancements regarding the effect of RE oxyhalides (REOCl) and oxyfluoride (REOF) in the in-depth deoxidation have been achieved by T.H. Okabe et al. [41], G. Kamimura et al. [42,43], A. Iizuka et al. [44,45], C.Y. Zheng et al. [46], L.X. Kong et al. [47,48], T. Tanaka et al. [49,50], L.G. Zhu et al. [51], H.Y. Fang et al. [52], and Z.Y. Tian et al. [53]. These studies investigated the deoxidation of solid/molten Ti and TiO_2_ in molten salt systems. In these systems, a RE-REOCl/REOF-RECl_3_/REF_3_ equilibrium was established, either by direct addition or situ reactions. The critical role lies in the formation of REOX (X = Cl or F) compounds, which possess significantly higher thermodynamic stability than their corresponding oxides (RE_2_O_3_). Even for REs with comparatively lower oxygen affinity, such as La and Ce, the generation of LaOCl or CeOCl results in a notable reduction in oxygen potential, substantially elevating the deoxidation limit. Moreover, REF_3_/REOF-based systems possess superior high-temperature stability relative to RECl_3_/REOCl, allowing them to remain effective above the melting points of refractory metals. This characteristic enables deoxidation in the molten state, greatly improving efficiency without compromising the thermodynamic limit—a crucial advantage for enhancing oxygen removal in high-melting-point metals. Representative deoxidation outcomes using REs are summarized in Table 1.

The speciation of REs during deoxidation, along with their consumption rate, is critically governed by the interplay between the oxygen potential and the chlorine potential within the system. While the studies discussed above have established the pivotal role of REOCl in enhancing the thermodynamic limit of deoxidation, several fundamental questions remain inadequately addressed and lack systematic elucidation. These include the synergistic variation mechanism of oxygen and chlorine potentials, their mutual coupling effects and the strategic regulation of oxygen potential in multi-element cooperative deoxidation systems.

In our previous work [38], a Ca-Y combined deoxidation system for Zr was established. By controlling the oxygen potential of the system, the thermodynamic mechanism for in-depth deoxidation based on Y-O solid solution and the equilibrium of Y-Y_2_O_3_ was elucidated. Building on this foundation, the present study systematically investigates the regulatory effect of chlorine potential on oxygen potential and the oxygen content of Zr. This research aims to clarify the synergistic interaction mechanism between oxygen and chlorine potentials and reveal the underlying principles of oxygen transfer and migration in a multi-element cooperative deoxidation system. Through the research, a theoretical framework is expected to be established for the construction of efficient, deep-level deoxidation systems, guiding process optimization.

## 2. Materials and Methods

### 2.1. Specimens Preparation

**Figure 2:**
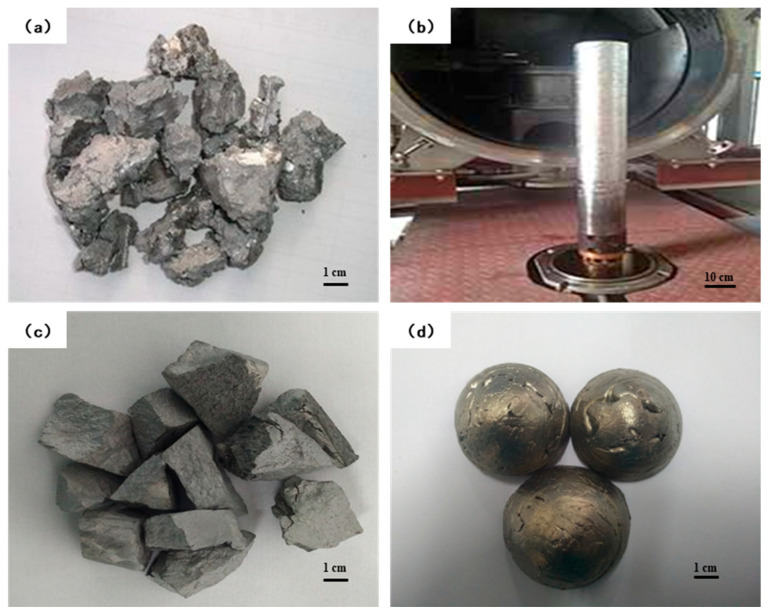
The raw materials and ingots of Y and Zr (**a**) sponge Zr; (**b**) EB melted Zr ingot; (**c**) Y lumps; (**d**) vacuum arc melted Y ingot.

The Zr specimens were prepared from nuclear-grade sponge Zr, which was purchased from State Nuclear BaoTi Zirconium Industry Co., Ltd., Baoji, China. The EB melting technique was employed to melt the nuclear-grade sponge Zr. After double melting, a Zr ingot with uniform composition was obtained. Then the ingot was subsequently cut into plates with a dimension of 12.5 × 12.5 × 2 mm, which were used as Zr specimens. The Y specimens were prepared from high-purity Y lumps, purchased from Grirem Advanced Materials Co., Ltd., Beijing, China, which were melted by a non-consumable vacuum arc melting process. The Y button ingot was sliced into plates with a dimension of 12.5 mm × 15 mm × 2 mm to serve as Y specimens. Figure 2 shows the raw materials and ingots of Y and Zr. The use of thin plates for both Zr and Y was intended to promote more homogeneous composition and reduce the time required for equilibration. Before the experiments, the Zr and Y specimens were polished to eliminate the surface oxide layer with 150 mesh sandpaper. The compositions and configurations of the Zr and Y specimens are provided in Table 2.

The reagents, e.g., Ca granules, anhydrous YCl_3_ powder and anhydrous CaCl_2_ powder, used in the experiments were reagent-grade and were purchased from Aladdin Reagents (Shanghai) Co., Ltd. (Shanghai, China). They were stored in a glove box with water and oxygen content both below 0.01 ppmw before use. The argon (Ar) gas used in this study was purchased from Beijing Praxair Industrial Gases Co., Ltd., Beijing, China, which has a purity of 5N. The typical composition of the Ar gas given in this report is shown in Table 3.

### 2.2. Experimental Procedures

**Figure 3:**
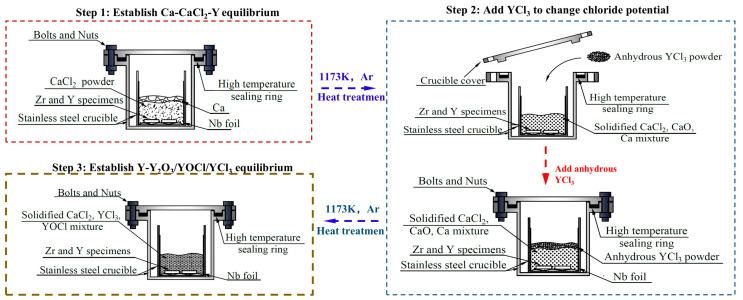
Schematic diagrams of the experimental procedures.

The experimental procedures of this study are primarily divided into three steps, as shown in Figure 3.

Step 1: Establishing Ca-CaCl_2_-Y equilibrium. A Zr specimen (~2.7 g), two Y specimens (~1.2 g each), 21 g of anhydrous CaCl_2_, and 3.5 g of metallic Ca granules were placed into a cylindrical 310S stainless steel crucible. To minimize the crucible’s influence on the reaction, its inner surface was first etched with a diluted HCl solution (H_2_O:HCl = 2:1, *v*/*v*) and then lined with Nb foil before use. This charge was designed to ensure complete submersion of all metallic specimens in the molten CaCl_2_ salt after melting. The loaded cylindrical crucible was then sealed within a reaction vessel consisting of a flanged assembly made of 310S stainless steel. The sealed reactor was subsequently placed in a resistance furnace, heated to 1173 K under a protective Ar atmosphere, and maintained at this temperature for 24 h. This duration was selected based on our previous work [54], which confirmed that the oxygen content reaches equilibrium under these conditions. Upon completion, the phase structure of the solidified mixture in the crucible achieved an equilibrium state of Ca-CaCl_2_-Y.

Step 2: Add YCl_3_ to change chlorine potential. After Step 1, the flanged reaction vessel was opened, and the inner cylindrical crucible was collected. YCl_3_ was then added into the cylindrical crucible in amounts equivalent to 50%, 80%, 110%, 130%, and 150% of the stoichiometric quantity required for its complete reaction with Ca, represented by Equation (1).

Ca (l) + 2/3 YCl_3_ (l) = CaCl_2_ (l) + 2/3 Y (s) (1)

Following the addition, the cylindrical crucible was reassembled into the flanged crucible and securely fastened with bolts and nuts.

Step 3: Establishing the Y-Y_2_O_3_/YOCl/YCl_3_ equilibrium. The reassembled crucible was placed in a resistance furnace and heated to 1173 K under an Ar atmosphere for varying durations. During this process, Ca and YCl_3_ will react according to Equation (1). Meanwhile, the excessive YCl_3_ will also react with Y_2_O_3_ to form YOCl as in Equation (2).

Y_2_O_3_ (s) + YCl_3_ (l) = 3YOCl (s) (2)

By the above reactions, the equilibrium of the system will shift from the Ca-Y-CaCl_2_ equilibrium (established in Step 1) toward a Y-Y_2_O_3_/YOCl/YCl_3_ equilibrium. After the reaction, the crucible was cooled to room temperature and removed from the furnace. The crucible cover was opened, and the molten salt was crushed and sampled for phase analysis. Subsequently, the residual molten salt was rinsed off with clean water, and the Zr specimens were recovered for oxygen content analysis. The interaction between oxygen potential and chlorine potential, as well as the mechanism of oxygen potential control, was then elucidated.

### 2.3. Analysis of Specimens

Oxygen content analysis: The oxygen content of the deoxidized and washed Zr and Y specimens was analyzed using the LECO ONH-836 analyzer (LECO Corporation, St. Joseph, MI, USA). The Zr and Y specimens were cut into strips approximately 2 mm in width. The surface impurities on the Zr specimens were removed in HF solution (HF/HNO_3_/H_2_O, 1:4:10, *v*/*v*) and rinsed with distilled water, ethanol, and acetone before being dried. The surface impurities on the Y specimens were removed by polishing with 400-grit sandpaper, using anhydrous ethanol as a coolant during the polishing process. The inert gas fusion-infrared absorption method was employed as the analysis method. The instrument employs a graphite crucible to hold the specimens, with a nickel basket serving as a fluxing agent. Before each measurement, three to five blank tests were performed, and the average value was used for blank compensation. The detection limit for oxygen was defined as three times the standard deviation of the blank, and the uncertainty of the blank value was at most ±20 ppmw. For each experimental condition, three independent specimens were analyzed, and the average oxygen content from the three specimens was taken as the result, with the standard deviation also calculated.

XRD analysis: The phase composition of the molten salt was analyzed by XRD. To ensure that the composition of the molten salt specimens can fully reflect the phase constitution of the molten salt, sampling should be carried out by crushing different positions within the crucible after the experiment. The recovered crushed salt specimens were ground into a homogeneous powder inside a glovebox. Phase composition analysis was performed using a D/max2550HB X-ray diffractometer (Rigaku Corporation, Tokyo, Japan) with Cu-Kα radiation (40 kV, 40 mA). The diffraction patterns were recorded over a 2θ range of 10° to 90° at a scanning speed of 5°/min. Phase identification was carried out using X’Pert HighScore software (version 2.2a, PANalytical B.V., Almelo, The Netherlands). The obtained spectral data were compared with standard PDF cards to identify the phase composition of the specimens.

## 3. Thermodynamic Considerations

Table 4 lists the standard Gibbs energies of different reactions used for thermodynamic analysis in this study.

**Figure 4:**
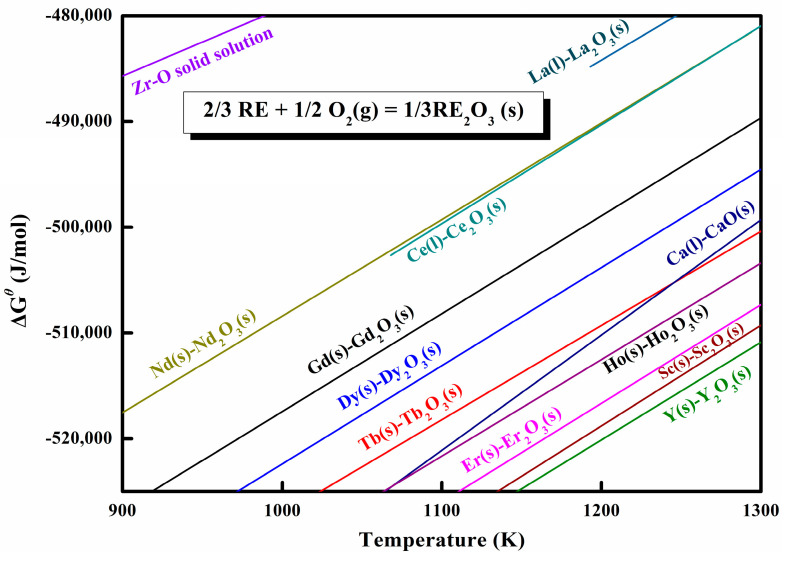
Comparison of the formation Gibbs energy of RE_2_O_3_, CaO and Zr-O solid solution.

The primary reason for selecting Y as a co-deoxidizing agent in this study is its exceptionally strong oxygen affinity. Figure 4 presents the oxygen potential diagram of various rare-earth metals. In this diagram, metals positioned lower exhibit stronger oxygen affinity. According to Figure 4, the order of oxygen affinity for the different rare-earth metals is as follows: Y (s) > Sc (s) > Er (s) > Ho (s) > Ca (l) > Tb (s) > Dy (s) > Gd (s) > Ce (l) > Nd (s) > La (l) > Zr-O (solid solution). Therefore, the use of yttrium enables effective regulation of calcium oxide activity in the molten salt and is thermodynamically most favorable for achieving deep oxygen removal. Although Sc, Er, and Ho can achieve similar effects, these rare-earth metals are expensive, and the extensive use of both the metals and their anhydrous chlorides would significantly increase the experimental cost.

**Figure 5:**
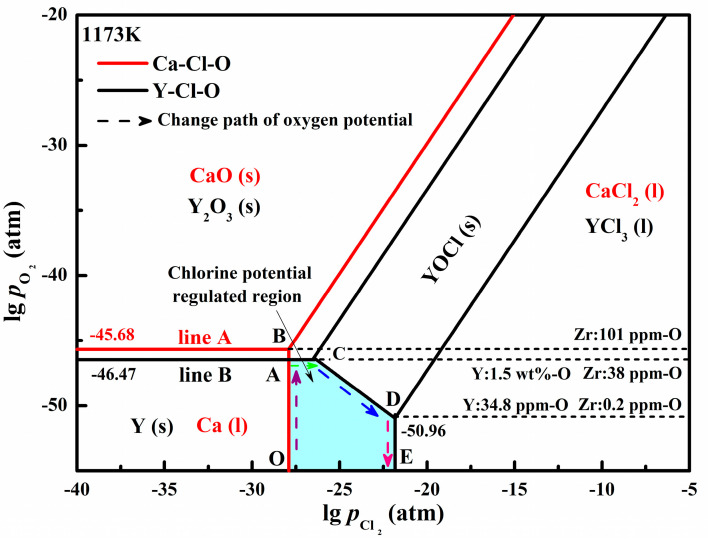
The superimposed oxygen–chlorine potential diagram of Y-Cl-O and Ca-Cl-O systems [38].

The oxygen–chlorine potential diagram is employed to illustrate the variation of oxygen partial pressure ($P_{O_{2}}$) with chlorine partial pressure ($P_{{\mathrm{Cl}}_{2}}$) during the Ca-Y combined deoxidation process. The superimposed $P_{{\mathrm{Cl}}_{2}}$-$P_{O_{2}}$ phase diagrams of Ca-Cl-O and Y-Cl-O systems at 1173K were plotted in Figure 5. The red and blue lines in Figure 5 constitute the thermodynamic stability regions of the Ca-Cl-O and Y-Cl-O systems, respectively. Each line corresponds to the relationship between oxygen potential and chlorine potential for a specific chemical reaction. For example, at the boundary line between the stability regions of CaO (s) and CaCl_2_ (l), by combining the reactions and Gibbs free energy expressions of Equations (18) and (19) in Table 4, Equations (25) and (26) are obtained,

CaO (s) + Cl_2_ (g) = CaCl_2_ (l) + 1/2O_2_ (g) (25)

Δ*G^θ^* = −157500 + 37*T* (26)

The actual Gibbs free energy expression Δ*G* for this reaction is given by Equation (27). When the reaction reaches thermodynamic equilibrium, ΔG = 0. Substituting Equation (26) yields Equation (28). Then, substituting the following parameters (*T* = 1173 K, *R* = 8.314 J/(mol·K), *p^θ^* = 101,325 Pa, *a*_CaO_ = 1, $a_{{\mathrm{CaCl}}_{2}}$ ≈ 1) into Equation (27), yields the relationship between the oxygen potential and chlorine potential of the system when CaO (s) and CaCl_2_ (l) coexist, as shown in Equation (28). The calculation of the other line equations follows the same method.

(27) $$-157500+37T=-RT\ln\frac{a_{CaCl_{2}}\cdot \sqrt{P_{O_{2}}}}{a_{CaO}\cdot P_{Cl_{2}}}$$

(28) $$\mathrm{lg}p_{O_{2}}=2\cdot \mathrm{lg}p_{Cl_{2}}+10.15$$

By simultaneously solving the Gibbs free energy expression for the Zr-O solid solution and those for the phase equilibrium lines in Figure 5, the corresponding equilibrium oxygen content in Zr can be calculated. Taking Line A as an example, by combining Equations (18) and (22) in Table 4, the reactions and Gibbs free energy expressions for calcium deoxidation of metallic zirconium are obtained, as shown in Equations (29) and (30),

O (in Zr) + Ca (l) = CaO (s)(29)

ΔG*^θ^* = −97423 + 44.7T (30)

When deoxidation reaches equilibrium, Δ*G* = 0, and Equation (31) is obtained,

(31) $$-97423+44.7T=-RT\ln\frac{a_{CaO}}{a_{Ca}\cdot {\left[O\right]}_{Zr}\cdot f}$$

Here, [O]_Zr_ represents the mass percent of oxygen in zirconium (with the 1 wt.% standard state), and *f* represents the activity coefficient of oxygen in zirconium. Previous studies [54] have demonstrated that within this oxygen concentration range, *f* = 1. Substituting the following parameters (*T* = 1173 K, *R* = 8.314 J/(mol·K), *a*_CaO_ = 1, *a*_Ca_ = 1, *f* = 1) into Equation (31), yields the [O]_Zr_ = 0.0101 wt.%, namely 101 ppmw. The calculation methods for the oxygen content of the other points are similar. The phase compositions and equations of the phase equilibrium lines are shown in Table 5.

As illustrated in Figure 5, at 1173 K, the theoretical equilibrium [O]_Zr_ corresponding to the Y-Y_2_O_3_ equilibrium (line B) is 38 ppmw, which is lower than the 101 ppmw corresponding to the Ca-CaO equilibrium (line A). The Y-Cl-O system exhibits a stable YOCl phase region. When YOCl, Y, and YCl_3_ coexist, the equilibrium oxygen potential of the system is located at point D, where the oxygen potential is extremely low, corresponding to a theoretical [O]_Zr_ of only 0.2 ppmw.

**Figure 6:**
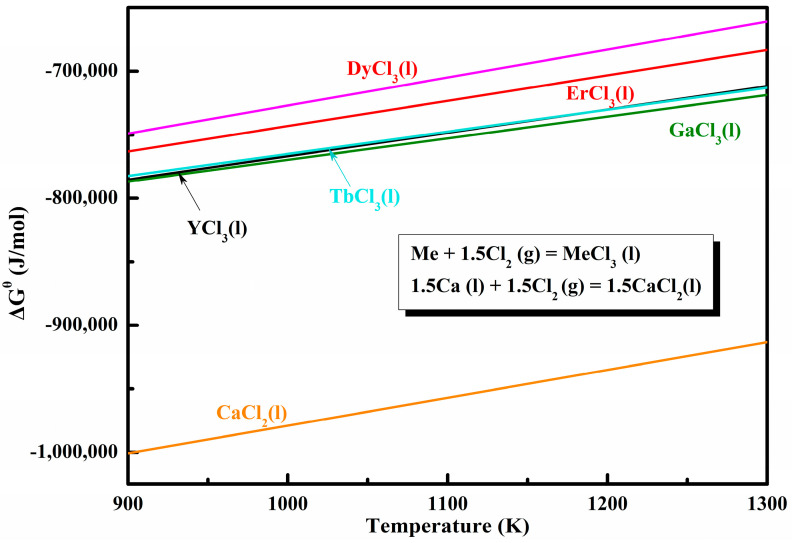
The Δ*G^θ^* for different metal chlorides [38].

The standard Gibbs energy of formation for different chlorides (CaCl_2_, YCl_3_, et al.) is compared in Figure 6.

Due to the significantly higher chlorine affinity of Ca compared to REs, Y can coexist with CaCl_2_ when immersed in a CaCl_2_ molten salt, whereas YCl_3_ would be reduced if in contact with Ca. Figure 6 also shows that the $P_{{\mathrm{Cl}}_{2}}$ corresponding to the Y-YCl_3_ equilibrium is considerably higher than that of the Ca-CaCl_2_ equilibrium, implying that the chlorine potential of the system can be modulated by the addition of YCl_3_. So, achieving point D in Figure 5 requires an increase in the chlorine potential of the system, which would result in the destabilization of Ca, leading to its complete conversion into CaCl_2_.

**Figure 7:**
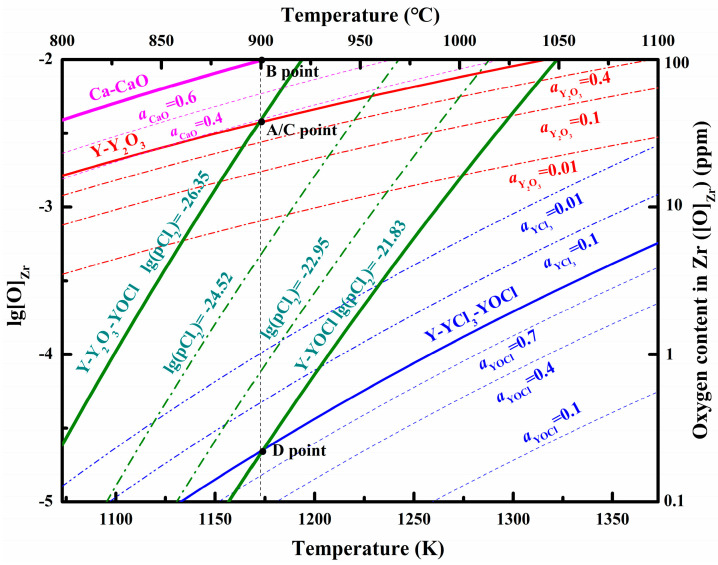
The relationship between [O]_Zr_ and *T*, *a*, $P_{{\mathrm{Cl}}_{2}}$.

The relationship between the [O]_Zr_ and *T*, *a*, and $P_{{\mathrm{Cl}}_{2}}$ under different equilibrium states is illustrated in Figure 7. By combining the equations for the different phase equilibrium lines in Figure 5 with the reaction formula and Gibbs free energy expression for the Zr-O solid solution, the oxygen content of zirconium metal under different equilibrium conditions can be calculated. Taking the Ca-CaO equilibrium as an example, rearranging Equation (31) yields Equation (32):

(32) $$\mathrm{lg}{\left[O\right]}_{Zr}\cdot f=\mathrm{lg}a_{CaO}-\mathrm{lg}a_{Ca}+\frac{-97423+44.7T}{2.303\cdot RT}$$

Substituting *f* = 1 and *a*_Ca_ = 1 into Equation (32) yields Equation (33), which establishes the relationship among [O]_Zr_, *T* and *a*_CaO_.

(33) $$\mathrm{lg}{\left[O\right]}_{Zr}=\mathrm{lg}a_{CaO}+\frac{-95088.12}{T}+2.33$$

By assigning values to *a*_CaO_, the relationships between the oxygen content of Zr and temperature under different *a*_CaO_ values are obtained. Table 6 summarizes the calculated equations for the oxygen content of Zr as a function of temperature, corresponding to various reactions and activity conditions.

As shown in Figure 7, the oxygen potential of the system decreases progressively with the sequential establishment of equilibria (Ca-CaO, Y-Y_2_O_3_, YOCl-Y, and Y-YCl_3_-YOCl) at different temperatures. Among them, the Y-Y_2_O_3_ equilibrium can reduce the *a*_CaO_ in the molten salt to below 0.4, thereby significantly enhancing the deoxidation capability of the Ca-CaO equilibrium. Meanwhile, the added YCl_3_ reacts with Y_2_O_3_ to form YOCl, decreasing the amount of $a_{Y_{2}O_{3}}$ in the molten salt to below 0.01, substantially improving the deoxidation capacity of the Y-Y_2_O_3_ equilibrium. Furthermore, with the formation of the YCl_3_ phase, the solubility of YOCl in the molten salt increases, leading to a further decrease in the *a*_YOCl_ and markedly strengthening the deoxidation effectiveness of the Y-YOCl equilibrium. Essentially, these changes reflect how the increase in chlorine potential continuously shifts the reaction equilibria during deoxidation: the activity of deoxidation products decreases step by step, which progressively raises the deoxidation capacity of the system.

**Figure 8:**
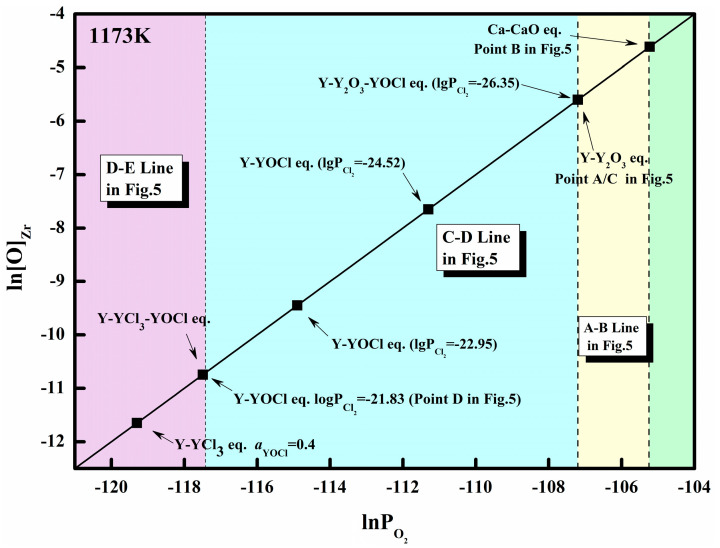
The relationship between the [O]_Zr_ and $P_{O_{2}}$ at 1173 K under different equilibrium states.

Figure 8 shows the relationship between the [O]_Zr_ and $P_{O_{2}}$ in the system at 1173 K under different equilibrium states. The straight line in Figure 8 represents the relationship between the oxygen potential and the oxygen content of the Zr–O solid solution at 1173 K. At thermodynamic equilibrium, Equation (34) can be derived from Equation (22):

(34) $$\frac{-543577+64.3T}{-RT}=\ln\frac{{\left[O\right]}_{Zr}\cdot f}{\sqrt{p_{O_{2}}}}$$

Substituting the parameters (*f* = 1, *T* = 1173 K, *R* = 8.314) into Equation (34) yields Equation (35), which corresponds to the straight line shown in Figure 8.

(35) $$\ln{\left[O\right]}_{Zr}=-\frac{1}{2}\cdot \ln p_{O_{2}}+48$$

Substituting the oxygen partial pressures calculated from the equations for different phase equilibrium relations in Table 6 into Equation (35) yields the corresponding oxygen contents in Zr at each point shown in Figure 8.

During the Ca-Y combined deoxidation process, as the chlorine potential of the system increases, the changes in $P_{O_{2}}$, [O]_Zr_ and the equilibrium state of the system can be divided into three stages, as depicted in Figure 5 and Figure 8:

(1) When the deoxidation system consists of Ca-CaCl_2_-Y, the equilibrium state lies on line OA. As deoxidation proceeds, the oxygen activity in molten salt increases, causing the equilibrium state to shift toward point A. During this stage, both the oxygen activity and the dissolved oxygen content in Y increase. Once the oxygen potential reaches point A, it will stabilize near this point, corresponding to the solubility of oxygen in Y. Any further oxygen absorption by Y will lead to the formation of Y_2_O_3_ and establishment of the Y-Y_2_O_3_ equilibrium that maintains the oxygen potential at a relatively low level. Only after the complete consumption of Y will the oxygen potential shift from point A to point B, at which point the Ca-CaO equilibrium takes over and governs the oxygen potential of the system. Throughout this process, the chlorine potential of the system remains constant at lg$P_{{\mathrm{Cl}}_{2}}$ = −27.9.
(2) At the thermodynamic equilibrium state corresponding to point A, the deoxidation system exhibits a four-phase coexistence of Y, Y_2_O_3_, Ca, and CaCl_2_. Increasing the chlorine potential of the system by introducing YCl_3_ induces a tendency for the equilibrium state to shift from point A toward point C. However, since the line OA represents the chlorination equilibrium of Ca-CaCl_2_, the increased chlorine potential leads to the chlorination of Ca. Once all the Ca in the molten salt is converted into CaCl_2_, the equilibrium state begins to shift from point A toward point C. Upon reaching point C, the chlorine potential of the system increases to lg$P_{{\mathrm{Cl}}_{2}}$ = −26.35 and the YOCl phase forms, resulting in the four-phase coexistence of CaCl_2_, Y, Y_2_O_3_, and YOCl.
(3) When the equilibrium of the deoxidation of the system is at point C, a further increase in the chlorine potential leads to the disappearance of the Y_2_O_3_ phase. Interestingly, this increase in chlorine potential is accompanied by a significant decrease in the oxygen potential. As a result, the equilibrium state shifts from point C toward point D. Ultimately, the system stabilizes at point D, which corresponds to an ultra-low oxygen potential region where Y, YOCl, YCl_3_, and CaCl_2_ coexist.

## 4. Results and Discussion

The oxygen content of Zr and Y specimens after step one deoxidation is shown in Table 7.

**Figure 9:**
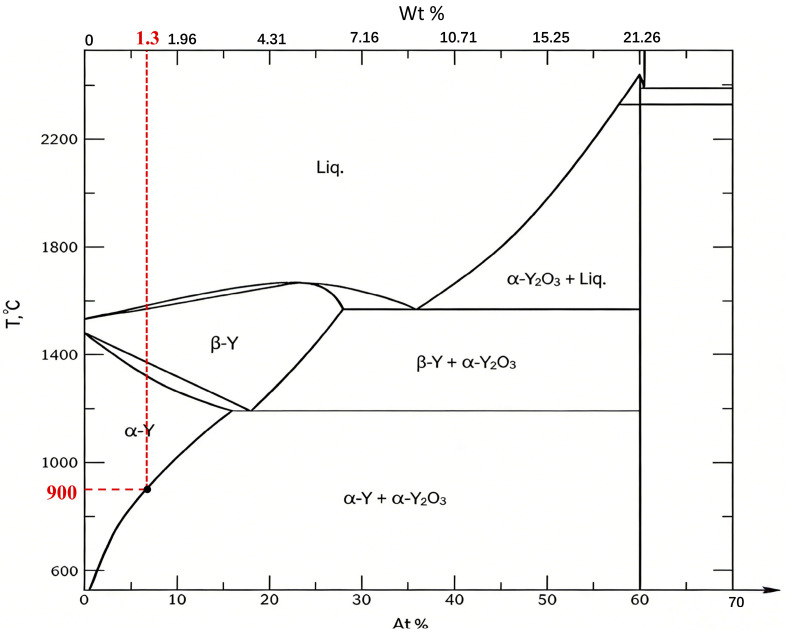
Y-O binary phase diagram and the solid solubility of oxygen in α-Y at 1173 K.

The [O]_Zr_ decreased from 800 ppmw to 48, 59, and 38 ppmw, and the [O]_Y_ increased from 0.5 wt.% to 1.02, 1.54, and 1.20 wt.%. Figure 9 presents the Y-O binary phase diagram. According to Figure 9, the solid solubility of oxygen in Y at 1173 K is approximately 1.3 wt.%.

The equilibrium [O]_Zr_ corresponding to the Y-Y_2_O_3_ equilibrium at 1173 K is 38 ppmw as depicted in Figure 5. Although no CaO was intentionally added to the experimental system, the [O]_Y_ after deoxidation still reached saturation, leading to the formation of Y_2_O_3_. This is primarily attributed to oxygen introduced by the raw materials Ca and CaCl_2_. For instance, a layer of CaO is inevitably present on the surface of Ca. Although nominally anhydrous, CaCl_2_ powder is highly hygroscopic and often contains trace amounts of moisture, CaO, or oxychloride. In addition, residual oxygen and water vapor in high-purity argon, as well as oxygen from the stainless steel crucible, may also be absorbed by Ca to form CaO. Under high-temperature conditions, these CaO and oxychloride can dissolve in the CaCl_2_ molten salt, releasing oxide ions, which are subsequently absorbed by Y, resulting in the formation of dissolved oxygen and the precipitation of Y_2_O_3_. Therefore, it can be concluded that after step one, the equilibrium state of the system has almost reached point A in Figure 5, corresponding to the Ca-CaCl_2_-Y-Y_2_O_3_ equilibrium.

**Figure 10:**
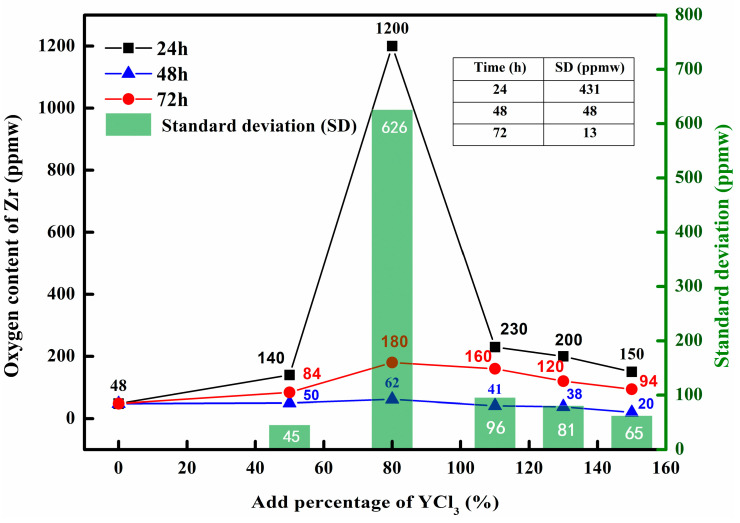
The dependence of equilibrium oxygen content on reaction time under different additions of YCl_3_.

Figure 10 shows the deoxidation results of Zr in the Ca-CaCl_2_-Y system with varying YCl_3_ additions (Step 2), respectively. The standard deviations (SD) of oxygen content for two dimensions, namely, different YCl_3_ additions under the same reaction time, and different reaction times under the same YCl_3_ addition, were also calculated and plotted in Figure 10 to reflect fluctuations in oxygen content.

To elevate the chlorine potential, YCl_3_ was added to the Ca-Y-CaCl_2_ system, which was initially at equilibrium point A in Figure 5, followed by a secondary equilibrium treatment at 1173 K. As can be seen from Figure 10, with the increase in YCl_3_ addition, the [O]_Zr_ exhibits significant fluctuations after 24 h of reaction. When the YCl_3_ addition is below 100%, the oxygen content increases sharply from 48 to 1200 ppmw, representing a 24-fold increase. Subsequently, as the YCl_3_ addition exceeds 100%, the oxygen content rapidly decreases to the range of 150–230 ppmw. When the reaction time is extended to 48 h, the extent of fluctuation of [O]_Zr_ is markedly reduced, although minor variations persist. Specifically, at a YCl_3_ addition of 80%, the [O]_Zr_ is 180 ppmw, representing a significant decrease of 85% compared to that observed at 24 h. Further prolonging the reaction time to 72 h results in nearly no fluctuation in [O]_Zr_, which instead displays a smooth and gradual decline. On the other hand, under the same reaction duration, a higher YCl_3_ addition leads to smaller fluctuations in [O]_Zr_. Under the same YCl_3_ addition, the SD of [O]_Zr_ at different reaction times more intuitively reflects the fluctuation of oxygen content during the process. As the YCl_3_ addition increases, the SD of oxygen content rises significantly from 45 ppmw at 50% addition to 626 ppmw at 80% addition. When the YCl_3_ addition exceeds 100%, the SD of oxygen content rapidly drops back to a level below 100 ppmw. For the SD values corresponding to different YCl_3_ additions at the same reaction time, as the reaction time increased from 24 h to 72 h, the SD decreased significantly from 481 to 13 ppmw, indicating a marked reduction in fluctuation.

**Figure 11:**
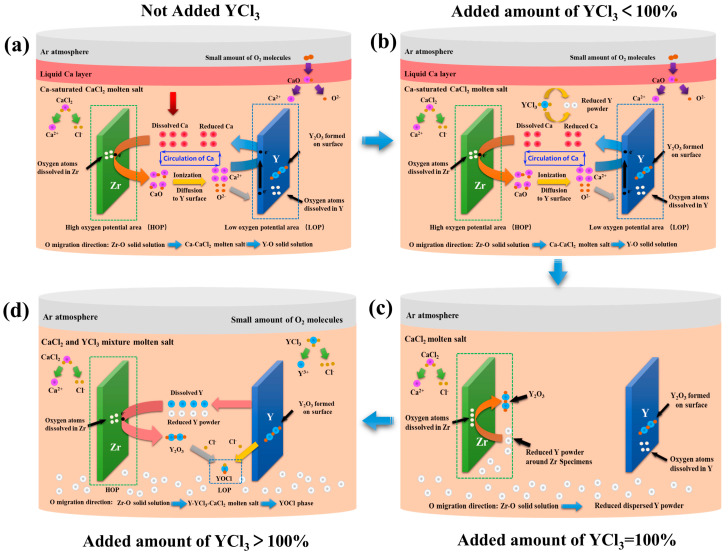
Schematic diagrams of deoxidation with varying YCl_3_ additions (**a**) without adding YCl_3_; (**b**) added an amount of YCl_3_ < 100%; (**c**) added an amount of YCl_3_ = 100%; (**d**) added an amount of YCl_3_ > 100% [38].

Figure 11 was employed to schematically illustrate the transition in oxygen potential control mode during the increase of chlorine potential in the system.

As shown in Figure 11a, when YCl_3_ was not added, the CaCl_2_ molten salt was saturated with liquid Ca. Although Y is insoluble in the CaCl_2_ molten salt, the dissolved Ca acts as an oxygen-transfer medium between Zr and Y specimens: it reacts with interstitial oxygen at the surface of Zr to form CaO, creating a high-oxygen-potential (HOP) zone in the molten salt. Then the formed CaO ionizes into the Ca^2+^ and O^2-^ in the CaCl_2_ molten salt. These ions diffuse to the Y surface, where Y absorbs the O^2-^ to form either a Y-O solid solution or Y_2_O_3_, establishing a low-oxygen-potential (LOP) zone in molten salt. The Ca^2+^ is subsequently reduced back to Ca, which re-enters the deoxidation cycle, thereby closing the Ca circulation loop. The presence of Ca significantly accelerates both the oxygen transfer rate at the metal–melt interface and the diffusion of oxygen through the molten salt.

When the added amount of YCl_3_ is less than 100%, as shown in Figure 11b, the liquid Ca was consumed to form Y powder, leading to a decrease in *a*_Ca_ and an increase in the oxygen potential of the system, thereby raising the [O]_Zr_. Due to the low solubility of Y and Y_2_O_3_ in CaCl_2_, the reduction in the amount of Ca results in deteriorated kinetic conditions during secondary equilibrium, necessitating a longer reaction time to re-establish the equilibrium state, although the oxygen potential of the entire system is intrinsically controlled by the Y-Y_2_O_3_ equilibrium. This indicates that Ca, dissolved in the CaCl_2_ molten salt, serves as a critical mass-transfer mediator during the deoxidation process: it facilitates the conversion of solid-solution oxygen into O^2-^ at the interface and accelerates oxygen transport between Zr and Y. Without this intermediary, despite the existence of a thermodynamic oxygen potential gradient, kinetic barriers will result in a significant reduction in deoxidation efficiency. These effects explain the sharp fluctuation in oxygen content observed after the addition of YCl_3_.

This deterioration in deoxidation kinetics is reversed when the addition of YCl_3_ exceeds 100%, as illustrated in Figure 11c,d. This manifests primarily in the following two aspects: (1) Reduction of oxygen potential via chemical reaction. Upon addition, YCl_3_ rapidly reacts with Y_2_O_3_ present in the molten salt to form YOCl. Since YOCl possesses a lower oxygen potential than Y_2_O_3_, this reaction effectively lowers the overall oxygen potential of the molten salt system, thereby promoting a decrease in [O]_Zr_. (2) Enhancement of Y solubility in the molten salt. The addition of YCl_3_ increases the dissolved amount of Y in the molten salt, which in turn intensifies the deoxidation process involving Y. When the YCl_3_ phase formed, the excess YCl_3_ and CaCl_2_ will form YCl_3_-CaCl_2_ binary molten salt, enhancing its solubility for Y and Y_2_O_3_. According to A.S. Dworkin et al. [59], the solubility of Y in YCl_3_ molten salt might be 5~10mol% (2.3~4.8 wt.%) at 1273 K. Although the solubility data at 1173 K are unknown, it can be predicted that YCl_3_ still possesses the ability to dissolve Y at this temperature, though this value is likely to be lower than that of Ca in CaCl_2_ at the same temperature (~6.5 wt.% [60]). This is a possible reason why the Ca-CaCl_2_ system kinetically outperforms the Y-YCl_3_ system. The dissolved Y will replace Ca, acting as the novel agent for oxygen transport, thereby improving the kinetic conditions of the secondary equilibrium. Consequently, a higher excess of YCl_3_ leads to smaller fluctuations and a lower [O]_Zr_, as illustrated in Figure 10. The oxygen potential control mechanism of the system shifts from the previous indirect Y-Ca combined mediation to direct control by Y. Overall, the equilibration time required for the indirect oxygen potential control via the Ca-Y-CaCl_2_ system (24 h) remains notably shorter than that for direct control via the Y-YOCl-YCl_3_ system (72 h).

The mass-transport characteristics can be semi-quantitatively evaluated to understand the kinetic bottleneck during the mediator transition. The diffusion flux (*J*) of the dissolved metallic mediator (M = Ca or Y) through the molten salt boundary layer governing the interfacial oxygen transport can be expressed by Fick’s First Law, represented by Equation (36):

(36) $$J=-D_{M}\cdot \frac{dc_{M}}{dx}\propto D_{M}\cdot \frac{C_{M,Bluk}-C_{M,Interface\;}}{\delta }$$

where *D*_M_ is the diffusion coefficient of the mediator (Ca or Y), *δ* is the effective boundary layer thickness, and *C*_M_ represents the local concentration. Maximally, the transport flux (*J*) is constrained by the thermodynamic solubility limit (*C*_sat._) of the mediator in the corresponding salt matrix (*J*_max_ ∝ *D*_M_·*C*_sat._).

In the initial Ca-CaCl_2_-Y system, liquid Ca exhibits a high solubility in molten CaCl_2_ at 1173 K. This elevated solubility sustains a large concentration gradient, driving a high flux (*J*_Ca_) that ensures rapid oxygen mass transfer across the melt, achieving equilibrium within 24 h. Conversely, when YCl_3_ addition is below 100%, Ca is progressively chlorinated and depleted. Because metallic Y is virtually insoluble in pure molten CaCl_2_, the active mediator concentration collapses (*C*_sat._ → 0), which severely restricts the overall diffusion flux. This sharp reduction in mass transport creates a kinetic barrier, preventing the system from reaching a homogeneous state within 24 h and manifesting as the massive fluctuations in [O]_Zr_ shown in Figure 10.

When the YCl_3_ addition surpasses 100%, the excess YCl_3_ blends with CaCl_2_ to form a binary mixed melt. This alters the solvent coordination and substantially enhances the solubility of metallic Y. The re-established solubility limit restores the mediator flux (*J*_Y_), thereby overcoming the kinetic delay and yielding smooth, low-fluctuation deoxidation profiles when the reaction time is extended to 72 h.

**Figure 12:**
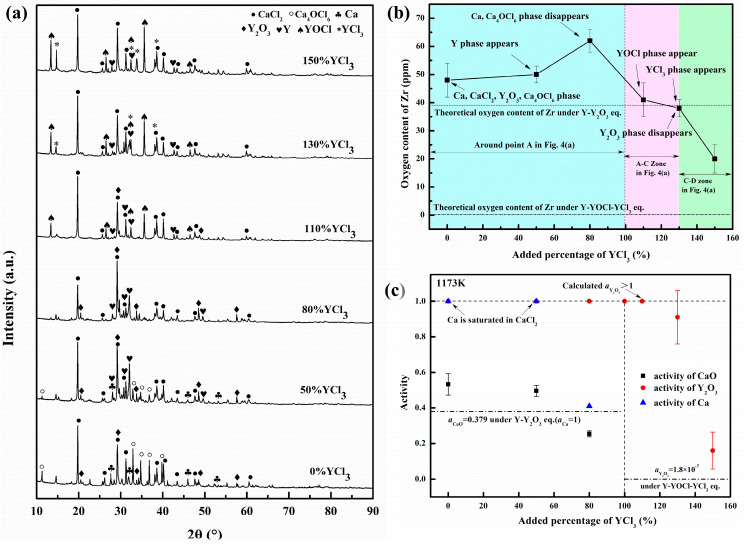
(**a**) Phase structure of the molten salt after 72 h of deoxidation with different YCl_3_ additions; (**b**) equilibrium oxygen content of Zr specimens with different YCl_3_ additions after 72 h of deoxidation; (**c**) the variation in *a*_Ca_, *a*_CaO_, and $a_{Y_{2}O_{3}}$ with different YCl_3_ additions after 72 h of deoxidation.

Figure 12a shows the phase structure of the molten salt after 72 h of deoxidation with different YCl_3_ additions. Figure 12b shows the variation in the [O]_Zr_ specimens with different YCl_3_ additions after 72 h of deoxidation.

As shown in Figure 12a,b, after Step 1, when no YCl_3_ was added, the main phases in the molten salt were CaCl_2_, Ca_4_OCl_6_, Ca, and Y_2_O_3_. The absence of the CaO phase indicates that the system equilibrium was controlled by the Y-Y_2_O_3_ equilibrium, which maintained the *a*_CaO_ at a low level, leading to the formation of the Ca_4_OCl_6_ phase. Under these conditions, the system equilibrium state lies on the OA line in Figure 5, corresponding to an oxygen content of 48 ppmw in Zr.

After additions of YCl_3_, the chlorination reaction of Ca occurred during the second equilibration process. The peaks corresponding to Ca gradually weakened, while those of Y strengthened. When the addition reached 80%, the Ca_4_OCl_6_ and Ca peaks almost disappeared. However, no YCl_3_ peaks were observed, and the peaks of Y_2_O_3_ remained strong. Throughout this process, the oxygen content in the Zr showed no significant change, indicating that the oxygen potential of the system remained essentially unaltered. When the amount of YCl_3_ added exceeded 110%, the peaks of YOCl significantly intensified, accompanied by a corresponding reduction in the intensity of the Y_2_O_3_ peaks. The equilibrium state of the system lies near point C. Once the YCl_3_ addition surpassed 130%, the Y_2_O_3_ peaks disappeared entirely, with minor peaks corresponding to YCl_3_ also observed. This indicates that Y_2_O_3_ in the molten salt had reacted completely with YCl_3_. Excessive YCl_3_ enters the molten salt, forming a mixed molten salt with CaCl_2_. When YCl_3_ addition exceeded 150%, the main phases present in the molten salt were CaCl_2_, YCl_3_, Y, and YOCl, forming a three-phase equilibrium state of Y-YCl_3_-YOCl, which corresponds to point D in Figure 5. During this process, the equilibrium [O]_Zr_ gradually decreased, ultimately reaching 20 ppmw. Although this value remains higher than the theoretical value of 0.2 ppmw corresponding to this point, a clear downward trend in oxygen potential has been established. This confirms that an increase in the chlorine potential of the system is indeed accompanied by a reduction in oxygen potential. It can be anticipated that if further YCl_3_ is added, dissolution of YOCl will occur, and the equilibrium oxygen potential of the system will move from point D toward point E, leading to a further decrease in oxygen potential.

The *a*_Ca_, *a*_CaO_, and $a_{Y_{2}O_{3}}$ were calculated using the oxygen content of Zr and the Gibbs energy equations of the corresponding reactions. The standard deviation and expanded uncertainty (*U*) of *a*_CaO_, and $a_{Y_{2}O_{3}}$ were also calculated and are shown in Table 8. The variation in activity of *a*_Ca_, *a*_CaO_, and $a_{Y_{2}O_{3}}$ during deoxidation is presented in Figure 12c.

It should be noted that the above activity calculations are based on the following approximations: (1) the *r*_Ca_ in the CaCl_2_ molten salt is assumed to be constant within the dilute solution range; (2) the oxygen dissolved in Zr is assumed to obey Henry’s law; and (3) the initial effect of YOCl formation on the activity of Y_2_O_3_ is neglected. These approximations are reasonable under conditions of low oxygen content ([O]_Zr_ < 100 ppmw) and moderate chlorine potential. However, when the YCl_3_ addition exceeds 130%, the calculated $a_{Y_{2}O_{3}}$ values become sensitive to the input parameters and should be regarded as order-of-magnitude estimates. Overall, this thermodynamic framework is suitable for revealing the trend of oxygen potential variation with chlorine potential, rather than providing precise absolute activity values. The uncertainties in the calculated activities arise from three main sources: (i) instrumental error in oxygen concentration measurement (repeatability: ±20 ppmw, as specified by the LECO ONH-836 analyzer); (ii) statistical variation among replicate samples (summarized as expanded uncertainty *U* with *k* = 2); and (iii) thermodynamic approximations, including the assumption of constant activity coefficient for Ca in molten CaCl_2_ and the Henrian behavior of oxygen in Zr-O solid solution. Even when considering the full uncertainty range, the key conclusions remain unchanged: The upper bound of a Y_2_O_3_ at 150% addition (0.16 + 0.12 = 0.28) is still substantially lower than 1 (the value corresponding to pure Y_2_O_3_ saturation). This confirms that Y_2_O_3_ is significantly consumed and YOCl forms as the chlorine potential increases.

As shown in Figure 12c and Table 8, when the addition of YCl_3_ is less than 100%, the oxygen potential of the system is controlled by the Y-Y_2_O_3_ equilibrium. When the YCl_3_ addition was ≤50%, Ca was in excess, and the Ca in the molten salt remained saturated. If the YCl_3_ addition is further increased to 100%, *a*_Ca_ drops to a lower level. This directly leads to a weakening of the deoxidation capacity of the Ca-CaCl_2_ system around the Zr specimens, resulting in the formation of a HOP zone. At this point, the Y-Y_2_O_3_ equilibrium acts as an “anchor” for the oxygen potential by reducing excess CaO in the molten salt, thereby lowering *a*_CaO_ and maintaining a low oxygen potential in the system. Due to the large contact area between Ca and YCl_3_ in the molten salt, the reaction between them proceeds rapidly. However, the small surface area of the Y specimens results in a relatively slow rate of CaO consumption via the Y-Y_2_O_3_ equilibrium. Consequently, the oxygen content of the Zr sample exhibits significant fluctuations in the initial reaction stage. Once the deoxidation equilibrium is reached, *a*_CaO_ decreases to match *a*_Ca_. Throughout this process, $a_{Y_{2}O_{3}}$ remains constant.

When the YCl_3_ addition exceeds 100%, the equilibrium state of the system is no longer dominated by the Y-Y_2_O_3_ equilibrium. Instead, YCl_3_ gradually consumes Y_2_O_3_ to form YOCl. During the variation of chlorine potential, the $a_{Y_{2}O_{3}}$ was calculated using the standard Gibbs energy expression for the Y-deoxidation reaction of Zr. At a YCl_3_ addition of 110%, Y_2_O_3_ is not fully consumed, and its activity remains at 1. When the YCl_3_ addition increases to 130%, Y_2_O_3_ is largely consumed, and its activity in the molten salt begins to decrease. Although the activity has not yet reached the theoretical value corresponding to the Y-YOCl-YCl_3_ equilibrium, the overall trend is consistent with the predictions of this equilibrium. During the entire process of chlorine potential regulation, the variation in the activities of various components in the molten salt follows similar trends to those shown in Figure 5 and Figure 12a,b.

## 5. Conclusions

High oxygen content has a significant effect on the properties of Zr. The Y-based solid-state deoxidation technology can effectively deoxidize Zr. However, the interaction mechanism of oxygen–chlorine potential and the control method of oxygen potential during the deoxidation process remain unclear. This study reveals the synergistic regulation mechanism between oxygen potential and chlorine potential in the Ca-Y-CaCl_2_ external gettering deoxidation system and clarifies the important role of dissolved Ca and Y in facilitating efficient interfacial transport of oxygen. These findings lay a foundation for the construction of efficient, deep-level deoxidation systems and provide critical guidance for process optimization. The main conclusions are as follows:

(1) Thermodynamic calculations indicate that oxygen potential decreases with increasing chlorine potential. As the chlorine potential increases from 1.27 × 10^−23^ Pa to 1.53 × 10^−17^ Pa, the phase constitution of the system progressively transitions from the initial Y-Ca-CaCl_2_ state to Y-Y_2_O_3_-CaCl_2_, then to Y-YOCl-CaCl_2_, and eventually to Y-YCl_3_-YOCl-CaCl_2_. At 1173 K, the theoretical [O]_Zr_ corresponding to the Y-YCl_3_-YOCl-CaCl_2_ system is only 0.2 ppm.
(2) For the equilibrated Ca-Y-CaCl_2_ system, YCl_3_ was added to regulate chlorine potential, followed by secondary deoxidation experiments over different durations. Oxygen content and molten salt phase constitution were characterized. Results show that after 24 h, [O]_Zr_ increased sharply from 48 to 1200 ppmw with increasing YCl_3_ addition, then dropped to 150–230 ppmw, showing a standard deviation (SD) of 431 ppmw. At 48 and 72 h, SD decreased to 48 and 12 ppmw, respectively. Regarding YCl_3_ addition, the maximum SD of 626 ppmw occurred at 80% addition, while below this, SD remained under 100 ppmw. After 72 h, the deoxidation process approached thermodynamic equilibrium, and the evolution of molten salt phase structure aligned with thermodynamic analysis. Ultimately, the [O]_Zr_ decreased to 20 ppmw at a YCl_3_ addition level of 150%.
(3) The Ca and Y dissolved in the molten salt serve as critical mass-transfer mediators during the deoxidation process: they facilitate the conversion of solid-solution oxygen into oxygen ions at the interface and enhance the migration of oxygen from high oxygen potential regions to low oxygen potential regions within the molten salt. In the absence of these mediators, even if a thermodynamic oxygen potential gradient exists, kinetic barriers will lead to a significant reduction in deoxidation efficiency.

While this study provides key insights, it is not without limitations. The ideal-solution assumption for the molten salt may not hold at high concentrations, and the kinetic analysis is primarily qualitative. As such, future work should progress to quantitative kinetic studies and more detailed phase characterization using advanced techniques such as SEM/EDS and TEM.

## Figures and Tables

**Table 1:** Representative deoxidation results in references using REs [18].

Target Metals	Equilibrium	Condition		Oxygen Content (ppmw)		Refs.
		Temperature (K)	Time (h)	Initial	After	
Zr	Y-Ca-CaCl_2_	1173	48	800	30~70	[38]
Ti *^a^*	Y-YF_3_-YOF	2000	0.16~0.33	1000	120~310	[41]
Ti	Ce-CeCl_3_-CeOCl	1200	48	120~1600	30~1600	[43]
Ti	Y-MgCl_2_-YCl_3_-(YOCl)	1300	48	230~1200	30~100	[44]
Ti	Y-MgCl_2_-YCl_3_-(YOCl)	1300	48	240~1160	600~3040	[46]
Ti	Ho-MgCl_2_-HoCl_3_-(HoOCl)	1173	48	240~1060	370~2180	[47]
Ti	Ho-HoCl_3_-HoOCl	1300	48	250~110	280~510	[48]
Ti *^b^*	La-LaCl_3_-LaOCl	1200~1300	24~48	200~1300	30~160	[49]
Ti *^b^*	La-MgCl_2_	1200	24~48	150~1300	210~3100	[50]
Ti *^b^*	Y-MgCl_2_-KCl	1073~1273	48	960, 1300	150~15,600	[51]
Ti	Y-YCl_3_-NaCl-KCl	1173~1273	48	1100, 1400	240~730	[52]

*^a^* Ti melt was deoxidized. *^b^* TiO_2_ was used as a raw material.

**Table 2:** Chemical analysis and configuration of specimens used in this study [38].

Specimen	Oxygen Content (ppmw)	Nitrogen Content (ppmw)	Carbon Content (ppmw)	Configuration (mm)
Zr	800	30	95	12.5 × 12.5 × 2
Y	5000	85	122	12.5 × 15 × 2

**Table 3:** The typical composition of the 5N-Ar provided by the supplier.

Index	Gas Composition/Mass Fraction ×10^−6^						
	H_2_	O_2_	N_2_	CO	CO_2_	CH_4_	H_2_O
Value	≤0.5	≤1.5	≤4.0	≤0.3	≤0.3	≤0.4	≤3.0

**Table 4:** The standard Gibbs free energies of different reactions used for thermodynamic analysis.

No.	Reactions	Standard Gibbs Energy (J/mol)	Temperature Range (K)	Refs.
(3)	2/3Gd (s) + 1/2O_2_ (g) = 1/3Gd_2_O_3_ (s)	Δ*G^θ^* = −610000 + 92.6*T*	900~1300	HSC (version 5.11)
(4)	2/3Tb (s) + 1/2O_2_ (g) = 1/3Tb_2_O_3_ (s)	Δ*G^θ^*= −616200 + 89.1*T*	900~1300	HSC (version 5.11)
(5)	2/3Dy (s) + 1/2O_2_ (g) = 1/3Dy_2_O_3_ (s)	Δ*G^θ^*= −615200 + 92.8*T*	900~1300	HSC (version 5.11)
(6)	2/3Er (s) + 1/2O_2_ (g) = 1/3Er_2_O_3_ (s)	Δ*G^θ^*= −628600 + 93.3*T*	900~1300	HSC (version 5.11)
(7)	2/3Ho (s) + 1/2O_2_ (g) = 1/3Er_2_O_3_ (s)	Δ*G^θ^* = −622100 + 91.3*T*	900~1300	HSC (version 5.11)
(8)	2/3La (s) + 1/2O_2_ (g) = 1/3La_2_O_3_ (s)	Δ*G^θ^* = −594800 + 91.6*T*	900~1193	HSC (version 5.11)
(9)	2/3La (l) + 1/2O_2_ (g) = 1/3La_2_O_3_ (s)	Δ*G^θ^* = −599800 + 95.9*T*	1193~1300	HSC (version 5.11)
(10)	2/3Ce (s) + 1/2O_2_ (g) = 1/3Ce_2_O_3_ (s)	Δ*G^θ^* = −599300 + 90.6*T*	900~1068	HSC (version 5.11)
(11)	2/3Ce (l) + 1/2O_2_ (g) = 1/3Ce_2_O_3_ (s)	Δ*G^θ^* = −602400 + 93.4*T*	1068~1300	HSC (version 5.11)
(12)	2/3Nd (s) + 1/2O_2_ (g) = 1/3Nd_2_O_3_ (s)	Δ*G^θ^* = −599700 + 91.3*T*	900~1294	HSC (version 5.11)
(13)	2/3Sc(s) + 1/2O_2_ (g) = 1/3Sc_2_O_3_ (s)	Δ*G^θ^* = −633000 + 95.2*T*	900~1300	HSC (version 5.11)
(14)	Gd (s) + 3/2Cl_2_ (g) = GdCl_3_ (l)	Δ*G^θ^* = −940000 + 170.3*T*	900~1300	HSC (version 5.11)
(15)	Tb (s) + 3/2Cl_2_ (g) = TbCl_3_ (l)	Δ*G^θ^* = −939300 + 174.2*T*	900~1300	HSC (version 5.11)
(16)	Dy (s) + 3/2Cl_2_ (g) = DyCl_3_ (l)	Δ*G^θ^* = −947500 + 220.5*T*	900~1300	HSC (version 5.11)
(17)	Er (s) + 3/2Cl_2_ (g) = ErCl_3_ (l)	Δ*G^θ^* = −942900 + 199.7*T*	900~1300	HSC (version 5.11)
(18)	Ca (l) + 1/2O_2_ (g) = CaO (s)	Δ*G^θ^* = −641000 + 109*T*	1100~1500	[55]
(19)	Ca (l) + Cl_2_ (g) = CaCl_2_ (l)	Δ*G^θ^* = −798500 + 146*T*	839~1484	[56]
(20)	Y (s) + 3/2Cl_2_ (g) = YCl_3_ (l)	Δ*G^θ^* = −941500 + 175.6*T*	900~1300	[57]
(21)	2/3Y (s) + 1/2O_2_ (g) = 1/3Y_2_O_3_ (s)	Δ*G^θ^* = −631000 + 92.4*T*	900~1300	[57]
(22)	1/2O_2_ (g) = O (in Zr)	Δ*G^θ^* = −543577 + 64.3*T*	1173~1373	[54]
(23)	1/2O_2_ (g) = O (in Y)	Δ*G^θ^* = −533000 + 12.4*T*	1177~1438	[35]
(24)	Y (s) + 1/2Cl_2_ (g) + 1/2O_2_ (g) = YOCl (s)	Δ*G^θ^* = −1075000 + 219*T*	1248~1357	[58]

**Table 5:** The phase compositions and equations of the phase equilibrium lines (see Figure 5).

Phase Composition	Reactions	Temperature/K	Equations
CaO (s)-CaCl_2_ (l)	CaO (s) + Cl_2_ (g) = CaCl_2_ (l) + 1/2O_2_ (g)	1173	$\mathrm{lg}p_{O_{2}}=2\cdot \mathrm{lg}p_{Cl_{2}}+10.15$
CaO (s)-Ca (l)	Ca (l) + 1/2O_2_ (g) = CaO (s)	1173	$\mathrm{lg}p_{O_{2}}=-45.68$
Ca (l)-CaCl_2_ (l)	Ca (l) + Cl_2_ (g) = CaCl_2_ (l)	1173	$\mathrm{lg}p_{Cl_{2}}=-27.90$
Y_2_O_3_ (s)-YOCl (s)	2Y_2_O_3_ (s) + 2Cl_2_ (g) = 4YOCl (s) + O_2_ (g)	1173	$\mathrm{lg}p_{O_{2}}=2\cdot \mathrm{lg}p_{Cl_{2}}+6.61$
YOCl (s)-YCl_3_ (l)	2YOCl + 2Cl_2_ (g) = 2YCl_3_ + O_2_ (g)	1173	$\mathrm{lg}p_{O_{2}}=2\cdot \mathrm{lg}p_{Cl_{2}}-7.3$
Y (l)-YOCl (l)	Y (s) + 1/2Cl_2_ (g) + 1/2O_2_ (g) = YOCl (s)	1173	$\mathrm{lg}p_{O_{2}}=-\mathrm{lg}p_{Cl_{2}}-72.8$
Y_2_O_3_ (s)-Y (s)	2/3Y (s) + 1/2O_2_ (g) = 1/3Y_2_O_3_ (s)	1173	$\mathrm{lg}p_{O_{2}}=-46.47$
Y (s)-YCl_3_ (l)	Y (s) + 3/2Cl_2_ (g) = YCl_3_ (l)	1173	$\mathrm{lg}p_{Cl_{2}}=-21.82$

**Table 6:** The relationship between the [O]_Zr_ and T under different reactions, activities and $p_{{\mathrm{Cl}}_{2}}$ (see Figure 7).

Equilibriums	Variables	Equations
Ca (l)-CaO (s)	*a*_CaO_ = 1.0	lg[O]_Zr_ = −11717.9/*T* + 5.37
	*a*_CaO_ = 0.6	lg[O]_Zr_ = −11717.9/*T* + 4.85
	*a*_CaO_ = 0.4	lg[O]_Zr_ = −11717.9/*T* + 4.45
Y (s)-Y_2_O_3_ (s)	$a_{Y_{2}O_{3}}$ = 1.0	lg[O]_Zr_ = −10515.1/*T* +3.38
	$a_{Y_{2}O_{3}}$ = 0.4	lg[O]_Zr_ = −10515.1/*T* +3.07
	$a_{Y_{2}O_{3}}$ = 0.1	lg[O]_Zr_ = −10515.1/*T* + 2.61
	$a_{Y_{2}O_{3}}$ = 0.01	lg[O]_Zr_ = −10515.1/*T* + 1.84
Y (s)-Y_2_O_3_ (s)-YOCl (s)	lg$p_{{\mathrm{Cl}}_{2}}$ = −26.35	lg[O]_Zr_ = −63919/*T* + 48.94
Y (s)-YOCl (s)	lg$p_{{\mathrm{Cl}}_{2}}$ = −24.52	lg[O]_Zr_ = −63919/*T* + 46.84
	lg$p_{{\mathrm{Cl}}_{2}}$ = −22.95	lg[O]_Zr_ = −63919/*T* + 45.02
	lg$p_{{\mathrm{Cl}}_{2}}$ = −21.83	lg[O]_Zr_ = −63919/*T* + 43.74
Y (s)-YCl_3_ (l)-YOCl (s)	$a_{YCl_{3}}$ = 0.01, *a_YOCl_* = 1.0	lg[O]_Zr_ = −26171/*T* + 13.12
	$a_{YCl_{3}}$ = 0.1, *a_YOCl_* = 1.0	lg[O]_Zr_ = −26171/*T* + 12.35
	$a_{YCl_{3}}$ = 1.0, *a_YOCl_* = 1.0	lg[O]_Zr_ = −26171/*T* + 11.59
	$a_{YCl_{3}}$ = 1.0, *a_YOCl_* = 0.7	lg[O]_Zr_ = −26171/*T* + 11.21
	$a_{YCl_{3}}$ = 1.0, *a_YOCl_* = 0.4	lg[O]_Zr_ = −26171/*T* + 10.65
	$a_{YCl_{3}}$ = 1.0, *a_YOCl_* = 0.1	lg[O]_Zr_ = −26171/*T* + 9.27

**Table 7:** The oxygen content of the specimens after step one deoxidation.

Steps	Oxygen Content of Specimens/ppmw	
	Zr	Y
Initial	800	5000
After Step One	48, 59, 38	1.02 wt.%, 1.54 wt.%, 1.20 wt.%

**Table 8:** Calculated *a*_Ca_, *a*_CaO_, and $a_{Y_{2}O_{3}}$ after reaction at 1173 K for 72 h under different YCl_3_ additions.

Add Amount of YCl_3_(%)	[O]Zr After 72 h Reaction/ppm			*a*_CaO_ *^a^*						$a_{Y_{2}O_{3}}$ *^b^*						*a*_Ca_ *^c^*
	[O]_1_	[O]_2_	[O]_3_	*a*_1_	*a*_2_	*a*_3_	avg.	SD	*U* (*k* = 2) *^f^*	*a*_1_	*a*_2_	*a*_3_	avg.	SD	*U* (*k* = 2) *^e^*	
0	48	59	38	0.48	0.59	0.38	0.49	0.11	0.13	/	/	/	/	/	/	1.0 *^d^*
50	52	50	46	0.52	0.50	0.46	0.50	0.03	0.035	/	/	/	/	/	/	1.0 *^d^*
80	64	64	57	0.26	0.26	0.23	0.25	0.02	0.023	/	/	/	/	/	/	0.41 *^e^*
110	45	44	38	/	/	/	/	/	/	1.70	1.59	1.02	1.44	0.36	0.42	/
130	40	39	34	/	/	/	/	/	/	1.00	1.00	0.73	0.91	0.15	0.17	/
150	24	21	14	/	/	/	/	/	/	0.26	0.17	0.05	0.16	0.10	0.12	/

*^a^* This value is calculated using Equation (32). Substituting the known parameters (*f* = 1, *T* = 1173K, *R* = 8.314 J/mol·K, [O]_Zr_, *a*_Ca_) into Equation (32) yields the expression: lg*a*_CaO_ = lg[O]_Zr_ − 2.00. Adopting the Raoultian standard state, the activity of pure solid CaO at 1173K equal to 1. *^b^* By combining the Gibbs energy expressions of Equations (21) and (22) in Table 4, and substituting the parameters (*f* = 1, *T* = 1173 K, *R* = 8.314 J/mol·K, [O]_Zr_, *a*_Y_ = 1), the expression for $a_{Y_{2}O_{3}}$ in the deoxidation reaction of Zr by Y is obtained: ln$a_{Y_{2}O_{3}}$ = 3ln[O]_Zr_ + 16.74. Adopting the Raoultian standard state, the activity of pure solid Y_2_O_3_ at 1173K equal to 1. *^c^* Adopting the Raoultian standard state, the activity of pure liquid Ca at 1173K equal to 1. *^d^* According to reaction Equation (1), the amount of Ca remaining after the reaction is still higher than its solubility in the CaCl_2_ molten salt, therefore *a*_Ca_ = 1. *^e^* Based on Equation (1), the consumed Ca was 2.8 g, the generated CaCl_2_ was 7.77g, leaving 0.77 g (0.018 mol) of residual Ca. The total mass of CaCl_2_ was 28.77(=21 + 7.77)g (0.26 mol), yielding a mole fraction of residual Ca of 0.063. The solubility of Ca in CaCl_2_ at 1173 K is 6.5 wt.%, corresponding to mole fraction *x*_Ca(sat.)_ of 0.153. Under the Raoultian standard state, *a*_Ca_ = 1 at saturation, yielding an *r*_Ca_ equal to 6.54 (*r*_Ca_ = 1/*x*_Ca(sat.)_ = 1/0.153 ≈ 6.54). Assume that the *r*_Ca_ is constant over the mole fraction range of 0.063–0.153, the *a*_Ca_ = 6.54 × 0.063 ≈ 0.41. *^f^* The standard uncertainty *u* = *SD*/(*n*^0.5^) (*n* = 3), and the expanded uncertainty *U* = *k*·*u* with *k* = 2, corresponding to a confidence level of approximately 95%.

## Data Availability

The original contributions presented in this study are included in the article. Further inquiries can be directed to the corresponding author.
